# The *Spodoptera exigua* ABCC2 Acts as a Cry1A Receptor Independently of its Nucleotide Binding Domain II

**DOI:** 10.3390/toxins11030172

**Published:** 2019-03-22

**Authors:** Daniel Pinos, María Martínez-Solís, Salvador Herrero, Juan Ferré, Patricia Hernández-Martínez

**Affiliations:** ERI de Biotecnología y Biomedicina (BIOTECMED), Department of Genetics, Universitat de València, 46100 Burjassot, Spain; daniel.pinos@uv.es (D.P.); maria.martinez-solis@uv.es (M.M.-S.); salvador.herrero@uv.es (S.H.); juan.ferre@uv.es (J.F.)

**Keywords:** mode of action, Sf21 cells, heterologous expression, truncated transporter, Bt resistance

## Abstract

ABC proteins are primary-active transporters that require the binding and hydrolysis of ATP to transport substrates across the membrane. Since the first report of an ABCC2 transporter as receptor of Cry1A toxins, the number of ABC transporters known to be involved in the mode of action of Cry toxins has increased. In *Spodoptera exigua*, a mutation in the *SeABCC2* gene is described as genetically linked to resistance to the Bt-product Xentari^TM^. This mutation affects an intracellular domain involved in ATP binding, but not the extracellular loops. We analyzed whether this mutation affects the role of the SeABCC2 as a functional receptor to Cry1A toxins. The results show that Sf21 cells expressing the truncated form of the transporter were susceptible to Cry1A toxins. Moreover, specific Cry1Ac binding was observed in those cells expressing the truncated SeABCC2. Additionally, no differences in the irreversible Cry1Ac binding component (associated with the toxin insertion into the membrane) were observed when tested in Sf21 cells expressing either the full-length or the truncated form of the SeABCC2 transporter. Therefore, our results point out that the partial lack of the nucleotide binding domain II in the truncated transporter does not affect its functionality as a Cry1A receptor.

## 1. Introduction

*Bacillus thuringiensis* (Bt) crystal proteins (Cry proteins) have been largely used in biological control as formulated sprays or in genetically-modified crops because of their high and specific toxicity against insect pests [1,2]. Due to the steady increase in the use of these proteins in agriculture, the appearance of resistance has been reported in some insect species, threatening the long-term use of Bt products [3].

Cry proteins are generally recognized as pore-forming toxins (PFTs), as their main action is to form pores in the membrane of the midgut epithelial cells of susceptible insects [4,5]. The mode of action of Cry proteins has been widely studied, especially for Cry1A proteins [6,7]. According with recent models, the mode of action of Cry proteins involves the sequential binding to different membrane receptors [7,8]. After specific binding events, the protein is inserted in the membrane inducing pore formation of the cells, which eventually leads to septicemia and insect death [7].

It is widely accepted that binding alteration is the most important mechanism of insect resistance to Cry toxins, although other mechanisms can also occur [3,9]. Alterations in the well-characterized midgut receptors for Cry toxins (cadherin-like protein, aminopeptidases N, alkaline phosphatases and ABC transporters) have been reported in different resistant strains [10,11,12,13]. In few cases, the expression of the receptor is altered, leading to the appearance of resistance [14,15,16,17,18]. In other cases, the receptors harbor different mutations that can alter the binding ability to Cry toxins [9,12,19,20,21].

ABC proteins are primary-active transporters that require the binding and hydrolysis of ATP to transport substrates across the lipid membrane. A functional ABC transporter consists of two cytosolic nucleotide-binding domains (NBDs) that bind and hydrolyze ATP, and two integral transmembrane domains, which generally consist of 5–6 transmembrane helices and provide substrate specificity [22]. To date, the number of ABC transporters that are involved in the mode of action of Cry toxins is increasing. The ABCC3 of *Spodoptera exigua* and *Spodoptera litura* and the ABCC4 of *Tribolium castaneum* were shown to be involved in susceptibility to Cry1A and Cry8C, respectively [23,24,25]. In addition, it has been observed that the ABCC2 acts as a functional receptor for Cry1A proteins for lepidopteran insects [26]. Interestingly, the ABCC2 from *S. exigua*, *Spodoptera frugiperda*, and *Bombyx mori* and the ABCC3 from *S. exigua* and *B. mori* do not function as functional receptors for Cry1C and Cry1D [21,25]. The implication of other ABC transporter subfamilies beyond subfamily C in the mode of action of different Cry proteins such as Cry2 and Cry3 has also been reported [18,27,28,29]. 

From the analysis of resistant strains to Bt proteins, different mutations in the *ABCC2* gene were reported to be associated with Cry1A resistance [13,30,31]. Furthermore, expression or silencing of the transporter using different systems correlates with altered susceptibility to Cry1 proteins [21,23,24,25,31,32,33,34,35], supporting the role of this family of transporters in the mode of action of Bt toxins. In agreement with that, binding assays have shown specific binding of Cry1A proteins to ABCC2 transporters from different insect species [32,36,37]. Additionally, it has been shown that ABCC2 transporters may also be involved in Cry1A oligomerization and/or insertion [34,37,38]. 

An early model for the binding of Cry1A toxins to the ABCC2 proteins suggested that the ABC transporter may interact with extended helices of the pre-pore Bt-toxin structure in its opened state and that, when it closes, an irreversible insertion is formed [39]. However, it was observed in a recent work in *B. mori* that lack of the ATPase activity (by generating NBD-deleted mutants) of the BmABCC2 transporter did not affect the functionality of the receptor variants [40].

In a laboratory-selected colony of *S. exigua* (Xen-R) resistant to the commercial Bt-product Xentari^TM^ [41], a mutation in the *SeABCC2* gene is described as genetically linked to the resistance [24]. In contrast to other mutations in *ABC* genes conferring resistance to Cry proteins, the mutation in *SeABCC2* affects an intracellular domain involved in ATP binding (NBDII). To determine whether this mutation affects both the ABCC2 function as a transporter and the Cry binding ability, the truncated form of the gene from the resistant strain (*SeABCC2-XenR*) was expressed in Sf21 cells. The functional role as receptor was tested by viability cell assays and the ability to bind Cry1A proteins was assessed.

## 2. Results and Discussion

### 2.1. Characterization and 3D Prediction of the Structure of the Truncated SeABCC2 Gene

**Figure 1:**
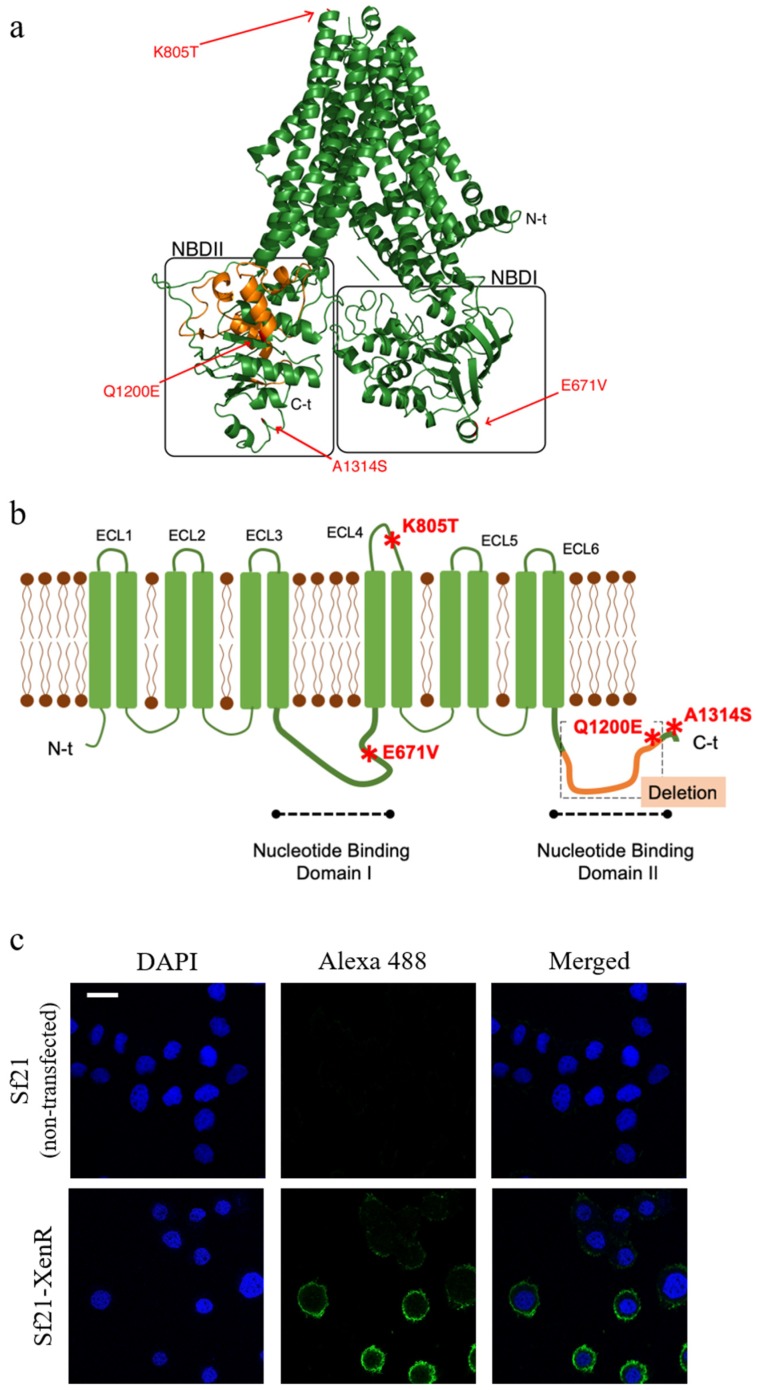
Predicted structure and expression analysis of the truncated SeABCC2. (**a**) Schematic illustration of the 3D structure of SeABCC2-FRA predicted using Phyre2 (http://www.sbg.bio.ic.ac.uk/phyre2/html/page.cgi?id=index) with reference to the 3D structure of atp-binding cassette sub-family C member 8 isoform X2. (**b**) 2-D schematic structure of the truncated SeABCC2 showing the ECL and NBD regions. The 1121–1199 amino acid deletion in the NBDII is shown in orange, and amino acid positions that differ in both proteins are shown in red. (**c**) Immunostaining of the SeABCC2-XenR transporter expressed in Sf21 cells. Cells were stained with an anti-FLAG tag antibody followed by anti-mouse IgG conjugated to Alexa Fluor 488 (green signal). Cell nuclei were stained with DAPI (blue signal). Scale bar represents 20 µm.

Park et al. [24] showed that the mutation in *SeABCC2* gene linked to resistance in the Xen-R colony was lacking part of NBDII, apparently not affecting the extracellular regions of the membrane protein. Compared with the wild type protein from the susceptible *S. exigua* colony (FRA colony) [41], the truncated transporter carries four additional single amino acid changes at positions 671, 805, 1200, and 1314 (Figure 1a,b). Based on the data obtained by the TMHMM server (which predicts transmembrane helices based on a hidden Markov model), one of these mutations (K805T) is located in the extracellular loop 4 (ECL4) of SeABCC2, whereas the other three are located in the intracellular part of the transporter (Figure 1b). Interestingly, the ECL4 region has been previously described as an important area for Cry1A toxin binding in BmABCC2 from *B. mori* [34,40]. 

### 2.2. The Truncated SeABCC2-XenR is Located in the Membrane of the Sf21 Cells

To examine whether single amino acid changes, along with the deleted NBDII region, are relevant for the interaction of Cry1A proteins and the SeABCC2 transporter, the truncated form was expressed in Sf21 cells. Expression of the *SeABCC2-XenR* gene was analyzed by RT-qPCR to determine whether the gene was stably expressed after transfection and selection. The expression levels were found significantly higher than the expression of the housekeeping gene, whereas essentially no expression was detected for the Sf21 cell line (non-transfected) (Figure S1a). Prior to test whether the truncated SeABCC2 transporter is a functional receptor for Cry1A proteins, its expression was determined by Western blot (Figure S1b). Moreover, the results from immunostaining showed that the truncated SeABCC2 transporter was located on the cell membrane of Sf21-XenR cells (Figure 1c). Therefore, despite harboring the deletion affecting the NBDII, the transporter was located in the Sf21 cell membranes, as reported previously for Sf21 cells expressing the full-length form of the transporter [37].

### 2.3. The Truncated SeABCC2 Still Acts as a Functional Receptor for Cry1A Toxins

Insertion/deletion (INDEL) mutations in the orthologous ABCC2 transporters have been reported to be associated with Cry1A resistance in different insect species [13,27,30,31,39]. However, direct evidence that these alterations on the ABC transporters are involved in Bt resistance by performing functional assays is scarce. Here, the functionality of the truncated ABCC2 molecule was studied. For this purpose, its role in toxicity was assessed in Sf21 cells expressing the SeABCC2-XenR.

**Figure 2:**
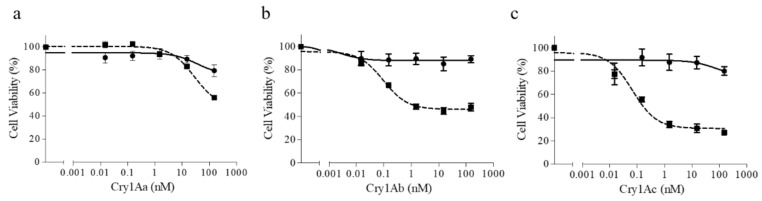
Effect of Cry1A proteins on the viability of Sf21 cells. Assays were performed using increasing concentrations of: Cry1Aa (**a**); Cry1Ab (**b**); and Cry1Ac (**c**). Assays were carried out for 3 h with Sf21 cells (circles) and Sf21-XenR cells (squares). Each value represents the mean of at least three independent assays (± SEM). Means were compared by two-way ANOVA, followed by Bonferroni’s comparison test.

The susceptibility of the two cell lines (Sf21 and Sf21-XenR) to three Cry1A proteins (Cry1Aa, Cry1Ab, and Cry1Ac) was determined by the MTT method. Regarding the Sf21 cells, none of the Cry1A toxins had any major effect on their viability, as shown by Martínez-Solís et al. [37] and Bretschneider et al. [32]. In contrast, the toxins affected the viability of Sf21-XenR cells in a dose-dependent manner. In addition, the loss of cell viability was drastic with Cry1Ab and Cry1Ac. For Cry1Aa, only significant differences were found at the highest concentration used (Figure 2). Similar results were found on Sf21-FRA cells, which express the full-length form of the transporter [37]. These results demonstrate that the ABCC2 transporter is necessary to render susceptibility to Cry1A toxins in Sf21 cells, independently of the presence of the second ATP binding domain. Thus, our results point out that the deletion on this domain is not directly causing resistance to the Cry1A type toxins in the Xentari^TM^-resistant *S. exigua* colony. Similarly, Tanaka et al. [40] found that cells expressing mutants of *B. mori* ABCC2 which lacked substrate-excreting activity still retained receptor activity for Cry1A toxins. Interestingly, cells expressing mutants with a deletion in the NBDII were susceptible to Cry1Aa and Cry1Ab but not to Cry1Ac. For *Helicoverpa armigera*, mis-splicing of the *ABCC2* gene was linked to Cry1Ac resistance [42]. The mis-splicing caused a 73 bp insertion that generated a premature stop codon, which expected to yield a truncated ABCC2 protein without the NBDII. However, based on our findings, functional analysis using the truncated HaABCC2 would be required to test if the truncation is causing the resistance to Cry1Ac in this strain or it is only partially contributing to resistance. More recently, a field-evolved resistance to Bt corn expressing Cry1Fa has been closely linked to a mutation in the *S. frugiperda ABCC2* gene [21]. The authors confirmed, by functional assays, that the full-length of the SfABCC2 acts as a functional receptor for Cry1Fa. In contrast, the mutated version of the SfABCC2 lacking the whole second transmembrane domain (consequently including the NBDII) was not functional.

### 2.4. The Truncated SeABCC2 Still Mediates Binding to Cry1A Toxins

**Figure 3:**
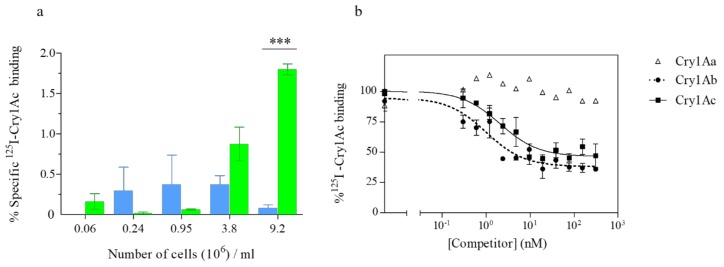
Binding of ^125^I-Cry1Ac to Sf21 cells expressing the SeABCC2-XenR. (**a**) Specific binding of ^125^ICry1Ac at increasing concentrations of Sf21 (blue) and Sf21-XenR (green) cells. Each bar represents the mean of at least three independent experiments (± SEM). Means were compared by two-way ANOVA, followed by Bonferroni’s comparison test (*p* <0.001). Significant differences between both cell lines are indicated by asterisks. (**b**) Competition binding assays with ^125^I-Cry1Ac using Sf21-XenR cells. Curves represent total binding of labeled Cry1Ac protein to increasing concentrations of unlabeled Cry1Aa (open triangles), Cry1Ab (full circles), and Cry1Ac (squares) as competitors. Each competition experiment was replicated at least three times and the error bars represent the standard error of the mean.

Binding assays using the ^125^I-labeled Cry1Ac protein were performed to verify the interaction of Cry1A proteins with the truncated transporter. The results showed specific binding of labeled Cry1Ac to increasing concentrations of the Sf21-XenR cells, while no specific binding was found for the control cell line (Sf21) at any given concentration (Figure 3a). Homologous competition assays showed that the competitor could completely displace the specific binding of labeled Cry1Ac to the Sf21-XenR cells (since there is almost 50% of nonspecific binding). Dissociation constant (*K*_d_) and concentration of binding sites (*R*_t_) were estimated from the homologous competition curve (Figure 3b), obtaining *K*_d_ = 2.4 ± 1.4 nM (mean ± SEM) and *R*_t_ = 0.006 ± 0.003 pmol/million cells (mean ± SEM). The *K*_d_ value indicates that binding of Cry1Ac to Sf21-XenR is of high affinity. The equilibrium binding parameter obtained in the present study did not differ significantly with the one obtained by Martínez-Solís et al. [37], when labeled Cry1Ac and cells expressing the full-length transporter were used. Interestingly, larger differences were found in the concentration of binding sites. Differences in the *R*_t_ values might be attributed to the difference in the expression levels of the transporter in each cell line. Therefore, the specific binding found for Sf21-XenR compared to the Sf21 cell line points out the fact that the intracellular truncation of the transporter does not affect the ability of the transporter to interact with the Cry1A toxins.

Different extracellular loops of ABCC transporters have been proposed as candidate Cry1A binding sites in different insect species [31,34,40,43]. In *B. mori*, it was reported that a single tyrosine insertion in the extracellular loop 2 (ECL2) causes resistance to Cry1Ab. Later, the same group showed that the amino acidic length of the ECL2 of the BmABCC2 is more important than the residues forming part of it. Furthermore, the authors stated that an increase in the length of the ECL2 disrupts the receptor function for Cry1Ab/c but not for Cry1Aa in the same species [34]. Here, we reported a mutation located in the ECL4 (position 805) of the truncated SeABCC2 transporter. This mutation, along with the other changes observed, was not affecting the role of the truncated transporter. Interestingly, it was recently reported in *B. mori* that the amino acid sequence from 770 to 773 of the ECL4 is a putative Cry1A toxin-binding site [40]. Liu et al. [43] reported that ABCC2 amino acid Q^125^ from SfABCC2 or E^125^ from SlABCC2 was a key factor for the differential Cry1Ac toxicity to Hi5 cells expressing these receptors. Interestingly, the authors claimed that, as these residues (Q^125^ or E^125^) are located in the ECL1 region of the ABCC2 transporter, this loop could be important for Cry1Ac binding. 

A deletion in the ABCC2 transporter of a Cry1Ac-resistant strain of *Plutella xylostella* (NO-QA strain) was genetically linked to resistance by Baxter et al. [30]. In this case, the deletion was predicted to remove the 12th transmembrane domain and aberrantly position the carboxyl-terminal outside the cell. Assuming that the gene is translated and inserted into the midgut membrane, the second ATP binding site was expected to be nonfunctional. Later, Hernández-Martínez et al. [20] demonstrated lack of Cry1Fa binding in the same *P. xylostella* strain but not to Cry1Aa. Lack of binding of Cry1Fa toxin was also observed on Sf9 cells expressing a mutated SfABCC2 transporter from a Cry1Fa-resistant strain of *S. frugiperda* [21]. The authors concluded that, based on the currently proposed model [39], the lack of binding of Cry1Fa could be due to the loss of the ATP-switch mechanism in the mutated transporter. However, it is important to highlight that the whole second transmembrane domain was absent in the mutated transporter including several ECL regions and the NBDII.

In the present study, heterologous competition experiments were performed in Sf21-XenR cells using Cry1Aa and Cry1Ab to determine whether these proteins shared binding sites with the Cry1Ac protein. The results showed that Cry1Ab competes against labeled Cry1Ac, while Cry1Aa does not compete (Figure 3b). Our results are in agreement with previous studies using *S. exigua* BBMV [44,45] and the Sf21-FRA cells [37]. Again, the results point out that the truncated SeABCC2 remains active as a functional receptor for the Cry1Ac and Cry1Ab toxins. 

### 2.5. The Irreversible Cry1Ac Binding Component is not Altered in the Truncated SeABCC2

**Figure 4:**
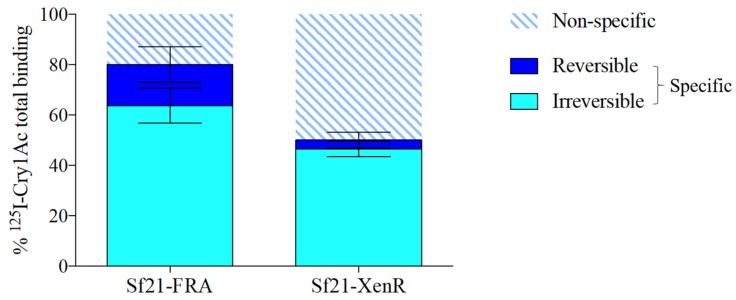
Dissection of total Cry1Ac binding into the non-specific and the specific binding (reversible and irreversible components) in Sf21 cells expressing the full-length (Sf21-FRA) or the truncated form (Sf21-XenR) of the SeABCC2 transporter.

The specific binding is considered as a critical step for the toxicity of Bt proteins [46]. Moreover, it is well established that specific binding of Cry toxins to their membrane receptors consists of a reversible and an irreversible binding component. The latter one has been associated with the toxin insertion into the membrane [47]. It has been suggested that the ABC transporters could be mediating the latter component [26,39]. Here, both components of the specific binding of Cry1Ac were determined in the Sf21-FRA and the Sf21-XenR cell lines to test whether the modifications found in the truncated transporter are affecting irreversible component in toxin insertion. The results showed that, for both cell lines, the predominant component of the specific binding was the irreversible binding (Figure 4). For Sf21-FRA cells, from the 80% of total specific binding, 64% was irreversible and 16% was reversible. For Sf21-XenR cells, from the 50% of total specific binding, 47% was irreversible and 3% was reversible. To test if the differences observed were significant, the proportion of irreversible binding on Sf21-FRA was compared to that in Sf21-XenR, as well as the proportion of reversible binding, finding no significant differences between the two receptors for the irreversible or the reversible binding (*p* = 0.1237, one-way ANOVA). Therefore, these findings, along with the viability assays, suggest that the Cry1Ac toxin can be inserted into the membrane despite the lack of the second ATP binding domain along with the other mutations found in the ABCC2. Recently, it was observed that pre-formed oligomers associate less efficiently with BBMV from the *P. xylostella* strain NO–QA (resistance linked to ABCC2) than with BBMV from a susceptible strain [38]. Interestingly, it is reported that a single tyrosine insertion in the ECL2 of the ABCC2 of *B. mori* causes resistance to Cry1Ab, although it can bind. Therefore, the authors suggested that the tyrosine insertion in the ECL2 may be affecting post-binding events [33]. Lastly, Park et al. [24] showed a significant decrease in the irreversible component of the Cry1Ca specific binding in *S. exigua* resistant to Xentari^TM^. This product is based on a *B. thuringiensis* subsp. *aizawai* (Valent Biosciences), containing Cry1Aa, Cry1Ab, Cry1C, Cry1D and Cry2Ab proteins. Since Cry1Ca is one of the most potent Cry toxins to *Spodoptera* spp. [48], this decrease in membrane insertion could contribute to the observed resistance against Xentari^TM^. 

## 3. Conclusions

The results from this study endorse that the truncated SeABCC2 (SeABCC2-XenR) transporter that lacks part of the NBDII is a functional receptor for the Cry1A proteins in *S. exigua.* In addition, the four additional single amino acid changes (positions 671, 805, 1200, and 1314) described here do not affect the functionality of the truncated receptor. Therefore, our data support that the ATP-switch mechanism of the transporter is not necessary to act as a functional receptor to Cry1A toxins. 

## 4. Materials and Methods 

### 4.1. Cell Culture Maintenance 

*Spodoptera frugiperda* derived Sf21 cells were cultured at 25°C in 25 cm^2^ tissue culture flasks (T25 flasks, Nunc) containing 4 mL of Gibco^®^Grace’s Medium (1×) (Life Technologies™, Paisley, UK) supplemented with 10% heat-inactivated fetal bovine serum (FBS). Sf21 cells stably expressing the wild type *SeABCC2* gene, designated as Sf21-FRA [37], were maintained at 27°C in the same culture medium supplemented with 10 μg/mL Blasticidin.

### 4.2. SeABCC2-XenR Structural Characterization

The amino acid sequence of the truncated form of *S. exigua* ABCC2 from the Xen-R colony (Acc. number AIB06822.1) was aligned with its wild type form (Acc. number AIB06821.1) using Geneious software (v10.2.6). The prediction of transmembrane domains, as well as the outer and inner parts of both forms of the SeABCC2 molecule were obtained using TMHMM server v 2.0 (http://www.cbs.dtu.dk/services/TMHMM/). The three-dimensional structure of SeABCC2-FRA was predicted using Phyre2 software (http://www.sbg.bio.ic.ac.uk/phyre2/html/page.cgi?id=index) with reference to the 3D structure of atp-binding cassette sub-family C member 8 isoform X2 as a template. 

### 4.3. Generation of Sf21 Clones Expressing SeABCC2-XenR

RNAzol RT reagent (Sigma-Aldrich, St. Louis, MO, USA) was used to isolate total RNA from midgut tissues of Xen-R colony larvae [41]. Then, total RNA was treated with DNase I (Invitrogen, Carlsbad, CA, USA) and reverse-transcribed to cDNA using specific primers (Table S1), which added *Sac*I and *Xba*I restriction sites as well as a FLAG-tag downstream in the gene. The cDNA encoding the truncated SeABCC2 (KF926100.1) form was cloned into the pIB-eGFP vector (kindly supplied by Monique Van Oers, Wageningen, The Netherlands) to generate the pIB-XenR vector. Sf21 cells were transfected with either pIB-eGFP or pIB-XenR vectors using the transfection reagent Cellfectin^®^II Reagent (Invitrogen). Selection was started 72 h post-transfection and the selective medium was replaced every 3–4 days until the cells reached confluence. Transfected cells were seeded in a T25 flask and maintained in Grace’s medium containing 10% FBS and 50 μg/mL Blasticidin (Invitrogen). The resulting polyclonal cell lines were named Sf21-eGFP and Sf21-XenR according to the vector used for transfection. Stable cell lines were maintained at 27 °C in Grace’s medium containing 10% FBS and 10 μg/mL Blasticidin.

### 4.4. RT-qPCR

The expression level of the *SeABCC2-XenR* gene in Sf21 cell lines was measured by RT-qPCR using specific primers (Table S1). After total RNA extraction from each cell line, cDNA was synthesized using PrimeScript RT Reagent kit (TaKaRa Bio Inc, Otsu Shiga, Japan). RT-qPCR was performed in a StepOnePlus Real-Time PCR System (Applied Biosystems, Foster City, CA, USA) following standard protocols. Primers used in this study were previously described and their efficiency tested by Martínez-Solís et al. [37]. The gene expression levels were normalized using the *ubiquitin* gene as a reference. 

### 4.5. Detection of the SeABCC-XenR Protein 

For the detection of the SeABCC2-XenR protein, Western blot was performed using monoclonal anti-FLAG^®^M2 (Sigma-Aldrich, St. Louis, MO, USA). First, membrane vesicle samples from both cell lines were prepared as described by van de Wetering et al. [49]. After SDS-PAGE, the samples were transferred to nitrocellulose membranes. Membranes were blocked with 3% of membrane blocking agent in PBST buffer (phosphate buffered saline with 0.1% Tween 20), and, after washing three times with PBST, membranes were incubated with monoclonal anti-FLAG^®^M2 (1:5000 dilution) in PBST supplemented with 1% blocking agent (PBST-B) for 1 h at RT. Then, membranes were washed three times with PBST and incubated with anti-mouse IgG-conjugated horseradish peroxidase (1:20,000 dilution) in PBST-B for 1 h at RT. Finally, bands were visualized with a chemiluminescence detection kit (RPN2209; GE Healthcare, Little Chalfont, UK) using an ImageQuant LAS4000 image analyzer (GE Healthcare).

For localization of the truncated transporter, immunostaining was performed as described by Martínez-Solís et al. [37]. Briefly, after overnight growing in 24-well glass chambers, cells were washed in phosphate buffered saline (PBS), fixed in 4% paraformaldehyde solution (PFA), permeabilized with 0.01% Triton X-100 at room temperature (RT), and blocked TNT buffer with 1% bovine serum albumin (BSA) for 1 h at RT. Cells were then incubated with monoclonal anti-FLAG^®^M2 (1:1000 dilution) for 2 h. After washes, cells were incubated with goat anti-mouse IgG conjugated to Alexa Fluor 488 (Abcam, Cambridge, UK) at a 1:1000 dilution for 1 h. Additionally, two negative controls were performed: (1) transfected cells were incubated with the secondary antibody alone; and (2) non-transfected Sf21 cells were incubated with the primary antibody alone. To stain the cell nuclei, 1 μg/mL of 4,6-diamidino-2-phenylindole (DAPI; Sigma Aldrich, Schnelldorf, Germany) was used. The stained samples on glass slides were observed under a confocal microscope (Olympus, FV1000MPE, Japan, Tokyo). 

### 4.6. Cry Proteins Preparation 

Cry1Aa, Cry1Ab and Cry1Ac toxins used in cell viability assays or binding assays were obtained from recombinant *Escherichia coli* or recombinant *B. thuringiensis* strains (from Ecogen Inc., Langhorn, PA, USA), respectively. The recombinant *E. coli* strains were kindly supplied by R. A. de Maagd. Purification of the inclusion bodies, Cry toxin solubilization and trypsin-activation was performed as described by Martínez-Solís et al. [37].

The Cry1Aa (EG1273), Cry1Ab (EG7077), and Cry1Ac (EG11070) toxins’ expression, solubilization, and trypsin-activation were performed as previously described by Estela et al. [50]. Trypsin-activated proteins were dialyzed in 20 mM Tris-HCl (pH 9) and filtered prior to anion-exchange chromatography using an ÄKTA system (GE Healthcare). 

The purity of all proteins was analyzed by sodium dodecylsulfate polyacrylamide gel electrophoresis (12% SDS-PAGE). All proteins were kept at −20°C until used.

### 4.7. Viability Assays 

Viability was determined after exposure of both cell lines (Sf21 and Sf21-XenR) to Cry1Aa, Cry1Ab and Cry1Ac toxins, using the MTT (3-[4,5-dimethylthiazol-2-yl]-2,5-diphenyltetrazolium bromide) assay. Prior to viability assays, cells were suspended in culture medium (without FBS) and plated (100 µL) in ELISA plates (flat bottom) at about 70% confluence. Plates were further incubated at 25 °C for at least 45 min to allow cells attach to the bottom. Then, 10 µL of activated toxins was added to each well within a range of increasing concentrations (from 0 to 150 nM) in duplicate on each plate. The same volume of 50 mM carbonate buffer (pH 10.5) and 2% Triton X-100 was also added to the wells as negative and positive controls, respectively. Cell viability was measured after 3 h incubation at 25 °C using the CellTiter 96^®^AQueous One Solution Reagent (Promega, Madison WI, USA) following the manufacturer’s protocol. After 2 h incubation, the absorbance was measured at 490 nm (Infinite m200, Tecan, Maennedorf, Switzerland). The percentage of viable cells was obtained as described by Martínez-Solís et al. [37]. For statistical analysis, means were compared by two-way ANOVA, followed by Bonferroni’s comparison test (*p* < 0.001).

### 4.8. Binding of ^125^I-Cry1Ac to Sf21 Cells

The purified and trypsin-activated Cry1Ac toxin was labeled using the chloramine-T method [51]. Briefly, Cry1Ac (25 μg) was mixed with 0.3 mCi of ^125^I (PerkinElmer, Boston, MA, USA), and 6 mM of chloramine T for 45 seconds at RT. After incubation, the reaction was stopped by adding sodium metabisulfite followed by sodium iodide (NaI). The specific activity obtained for the labeled Cry1Ac toxin was 15 μCi/μg.

All binding assays were conducted at RT in a final volume of 0.1 mL in binding buffer. Firstly, both cells lines (Sf21 and Sf21-XenR) were recovered by centrifugation (500× *g* for 5 min at RT), and washed two times with PBS. The cell pellet was resuspended in binding buffer (PBS supplemented with 0.1% BSA). to a concentration of 4.6 × 10^7^ cells/mL. The optimal concentration of cells to be used for the binding assays was calculated with increasing amounts of cells incubated with 0.1 nM of labeled-Cry1Ac in binding buffer. An excess of unlabeled protein (150 nM) was added to the reaction mixture to determine the nonspecific binding. After incubation, samples were centrifuged (500× *g* for 10 min) and the pellets were washed with binding buffer. Final radioactivity was measured in a gamma counter (2480 WIZARD^2^). Binding experiments were performed at least twice for each cell line.

Competition experiments were performed incubating the Sf21-XenR cells (9.2 × 10^6^ cells/mL) with ^125^I-Cry1Ac and increasing amounts of different unlabeled Cry1Aa, Cry1Ab or Cry1Ac proteins. After incubation, samples were centrifuged, washed, and radioactivity in the final pellets measured. Competition assays were replicated at least three times. The equilibrium dissociation constant (*K*_d_) and concentration of binding sites (*R*_t_) were obtained from the homologous competition experiments using the LIGAND software [52].

The contribution of reversible and irreversible binding to the observed specific binding in those cells expressing either the full-length or the truncated form of the SeABCC2 molecule was determined as described by Park et al. [24]. Briefly, three reaction mixtures were prepared with 0.1 nM of ^125^I-Cry1Ac and 9.2 × 10^6^ cells/mL of either Sf21-FRA or Sf21-XenR cell lines. The first sample was used to determine the total binding. In the second sample, an excess of unlabeled Cry1Ac toxin (150 nM) was added to this mixture at the beginning of the incubation to determine the non-specific binding. Finally, the third sample was used to measure the irreversible binding. To that purpose, after one hour of incubation, an excess of unlabeled Cry1Ac toxin was added and the reaction was allowed to proceed one more hour. All samples were incubated at RT for two hours. The specific and the irreversible binding were calculated by subtracting the non-specific binding (radioactivity in the pellet of the second sample) from the total binding (radioactivity in the first sample) or from that in the third sample, respectively. The reversible component was calculated by subtracting the irreversible binding from the specific binding. Experiments were performed three times.

## Supplementary Materials

The following are available online at https://www.mdpi.com/2072-6651/11/3/172/s1, Figure S1: Detection of the truncated ABCC2. (**a**) Expression levels measured by RT-qPCR in Sf21 and Sf21-XenR insect cell lines. The *ubiquitin* gene was used as housekeeping gene. The gene expression is given as copy number per 1000 molecules ubiquitin ± SEM. Means were compared by t-test (*p* <0.01). Significant differences are indicated by asterisks. (**b**) Western blot analysis showing the presence of the truncated SeABCC2 transporter (black arrow, *ca*. 142 kDa) in the membrane vesicles of the cells of Sf21 and Sf21-XenR cells. First line, molecular mass marker (in kDa). Table S1: Sequence of the primers used in this study.
