# Supplementary material for: The Spodoptera exigua ABCC2 Acts as a Cry1A Receptor Independently of its Nucleotide Binding Domain II
Source: Toxins (Basel). 2019 Mar 22;11(3):172. doi: 10.3390/toxins11030172 (PMC6468857; doi:10.3390/toxins11030172)
Supplement: Supplementary file 1 [file toxins-11-00172-s001.pdf]

toxins

Supplementary Materials: The *Spodoptera exigua* ABCC2 Acts as a Cry1A Receptor Independently of its Nucleotide Binding Domain II

Daniel Pinos, María Martínez-Solís, Salvador Herrero, Juan Ferré and Patricia Hernández-Martínez

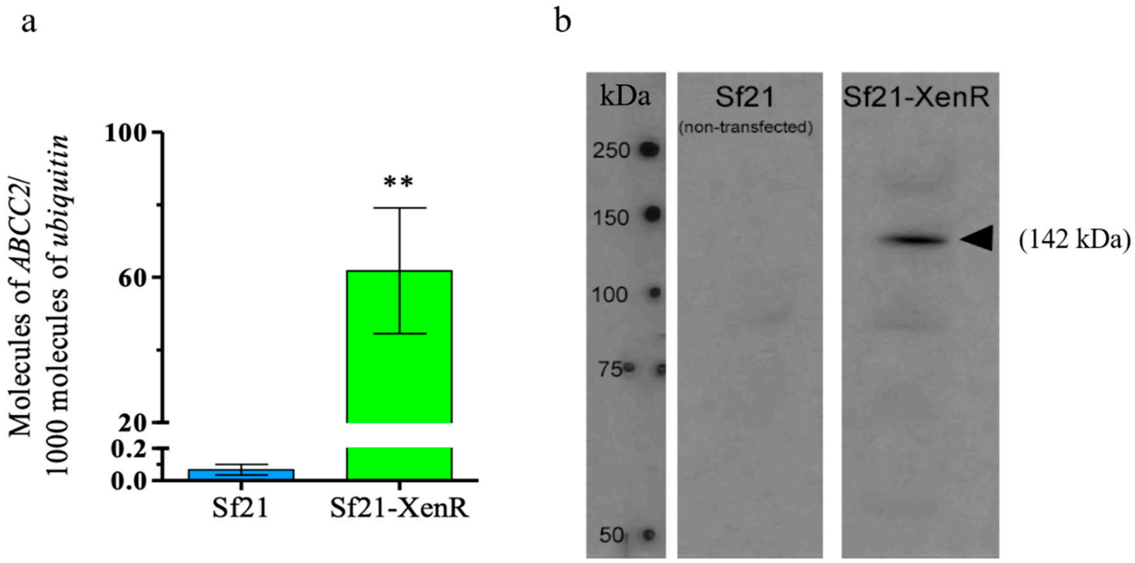

Figure S1. Detection of the truncated ABCC2 (**a**) Expression levels measured by RT-qPCR in Sf21 and Sf21-XenR insect cell lines. The *ubiquitin* gene was used as housekeeping gene. The gene expression is given as copy number per 1000 molecules ubiquitin ± SEM. Means were compared by T-test (p <0.01). Significant differences are indicated by asterisks. (**b**) Western blot analysis showing the presence of the truncated SeABCC2 transporter (black arrow, *ca.* 142 kDa) in the membrane vesicles of Sf21 and Sf21-XenR cells. First line, molecular mass marker (in kDa).

Table S1. Sequence of the primers used in this study.

Primer name	Sequence (5′–3′)
Cloning	
SeABCC2_SacI[F]	CGAGCTCATGGACAAATCGAATAAA
SeABCC2_FLAG/XbaI[R]	GTCTAGACTACTTGTCGTCATCGTCTTTGTAGTCAGCGGTTTTGGAATCACTTT
qRT-PCR	
qF_SeABCC2	AGCTACCGACCGAGGAAAAT
qR_SeABCC2	CTCTCCAGCACTAGGCCATC
qF-ubiquitin	GTTGCTGGTCTGGTGGGATT
qR-ubiquitin	AGGCCTCAGACACCATTGAAA
